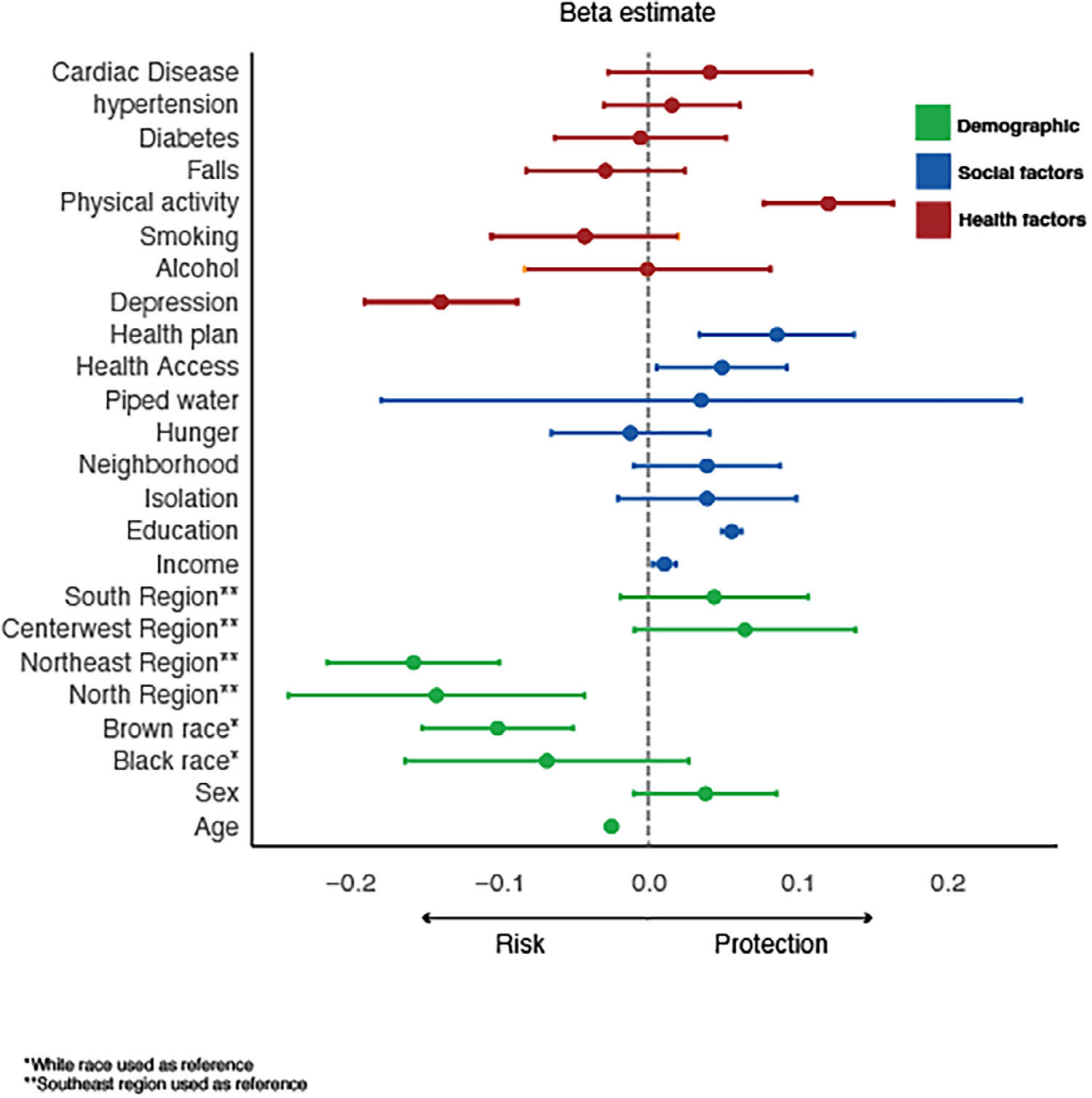# Modifiable and non‐modifiable disparity‐related predictors of cognitive scores in a population‐based cohort: the ELSI‐Brazil

**DOI:** 10.1002/alz.092264

**Published:** 2025-01-09

**Authors:** Wyllians Vendramini Borelli, Lucas Uglione Da Ros, Cristiano Schaffer Aguzzoli, Marco Antônio de Bastiani, Claudia Kimie Suemoto, Eduardo R. Zimmer

**Affiliations:** ^1^ Federal University of Rio Grande do Sul, Porto Alegre, Rio Grande do Sul Brazil; ^2^ Memory Center, Hospital Moinhos de Vento, Porto Alegre, RS Brazil; ^3^ Universidade Federal Do Rio Grade Do Sul, Porto Alegre, Rio Grande do Sul Brazil; ^4^ Brain Institute of Rio Grande do Sul, Porto Alegre, RS Brazil; ^5^ Division of Geriatrics, University of São Paulo Medical School, São Paulo, São Paulo Brazil; ^6^ Universidade Federal do Rio Grande do Sul, Porto Alegre, Rio Grande do Sul Brazil; ^7^ McGill University, Montreal, QC Canada; ^8^ Brain Institute of Rio Grande do Sul ‐ Pontifícia Universidade Católica do Rio Grande do Sul, Porto Alegre, Rio Grande do Sul Brazil

## Abstract

**Background:**

Social and health‐related disparity factors are important predictors of brain health in low and middle‐income countries (LMIC). Social predictors of cognition have a higher impact on brain health among LMICs than classic demographic factors, such as age and sex. This study aimed to evaluate the impact of modifiable and non‐modifiable social and health‐related factors on cognition in a Brazilian population‐based cohort.

**Method:**

We selected 9,412 individuals from the ELSI‐Brazil cohort, which is a population‐based study conducted with a similar design as the Health and Retirement Study. Complex sample design stratified and clustered distinct geographical regions according to their representation nationally. We included health‐related disparity factors (hypertension, diabetes, cardiovascular disease, and falls), as well as social determinants of health (piped water, access to healthcare, private health coverage, income, food insecurity, and education). Non‐modifiable factors were also retrieved (age, sex, race and region). We evaluated cognition using a global composite of orientation, animal fluency, and memory recall scores. A linear regression model was conducted to identify and stratify predictors of global cognitive scores (R2 adjusted = 0.336).

**Result:**

We included 5,432 individuals above 60 years of age in this analysis (mean age 70.3±7.97 yo). The regression model identified that the most important modifiable predictor of cognition was depression (ß = ‐0.14±0.06, *p < 0.001*), followed by physical activity (ß = 0.12±0.03, *p < 0.001*), and private health coverage (ß = 0.09±0.06, *p = 0.001*). Among non‐modifiable predictors of cognition, northeast region dwelling was the most important predictor (ß = ‐0.16±0.05, *p < 0.001*), following residing in the north region (ß = ‐0.14±0.10, *p = 0.006*), and brown race (ß = ‐0.1±0.05, *p < 0.001*). No health‐related factor reached significance.

**Conclusion:**

Overall, modifiable and non‐modifiable social‐related disparity factors had a significant impact on cognition in this study. Modifiable factors should be addressed in public health policies, while non‐modifiable risk factors may provide insights on compensatory strategies to overcome their negative effects on brain health. Further studies may expand this investigation to other LMIC.